# Supplementary material for: Empirical methods for controlling false positives and estimating confidence in ChIP-Seq peaks
Source: BMC Bioinformatics. 2008 Dec 5;9:523. doi: 10.1186/1471-2105-9-523 (PMC2628906; doi:10.1186/1471-2105-9-523)
Supplement: Additional File 1 — A variety of html documents from the USeq web site detailing the available applications, their best usage, output file type descriptions, command line menus, etc. [file 1471-2105-9-523-S1.zip › USeqUserGuides/JavaDoc.zip!/JavaDoc/util/bio/cluster/Cluster.html]

Cluster


|  |  |  |
| --- | --- | --- |
| **Overview** \| **Package** \| **Class** \| **Use** \| **Tree** \| **Deprecated** \| **Index** \| **Help** | |  |
| PREV CLASS   **NEXT CLASS** | **FRAMES**    **NO FRAMES**     **All Classes** |  |
| SUMMARY: NESTED \| FIELD \| CONSTR \| METHOD | DETAIL: FIELD \| CONSTR \| METHOD |  |


---


## util.bio.cluster Class Cluster

```
java.lang.Object
  util.bio.cluster.Cluster
```

**All Implemented Interfaces:**: java.io.Serializable

---

``` public class Cluster extends java.lang.Object implements java.io.Serializable ```

**See Also:**: Serialized Form

---

**Constructor Summary**

`Cluster(Cluster one,
Cluster two,
double correlationCoefficient,
java.lang.StringBuffer results)`
  
          Secondary Constructor

`Cluster(java.io.File floatArrayFile,
java.lang.String name)`
  
          Initializing Constructor


| **Method Summary** | |
| --- | --- |
| `Cluster` | `getBottomCluster()` |
| `double` | `getCorrelationCoefficient()` |
| `java.io.File` | `getFloatArrayFile()` |
| `java.awt.geom.Line2D` | `getLine()` |
| `java.lang.String` | `getName()` |
| `javax.swing.JLabel` | `getNameLabel()` |
| `double` | `getNameLabelCenterYCoordinate()` |
| `Cluster` | `getParentCluster()` |
| `java.awt.geom.Point2D` | `getParentPoint()` |
| `Cluster` | `getTopCluster()` |
| `double` | `getTotalClusters()` |
| `float[]` | `getValues()` |
| `void` | `setBottomCluster(Cluster bottomCluster)` |
| `void` | `setCorrelationCoefficient(double correlationCoefficient)` |
| `void` | `setFloatArrayFile(java.io.File floatArrayFile)` |
| `void` | `setLine(java.awt.geom.Line2D line)` |
| `void` | `setName(java.lang.String name)` |
| `void` | `setNameLabel(javax.swing.JLabel nameLabel)` |
| `void` | `setNameLabelCenterYCoordinate(double centerYCoordinate)` |
| `void` | `setParentCluster(Cluster parentCluster)` |
| `void` | `setParentPoint(java.awt.geom.Point2D parentPoint)` |
| `void` | `setTopCluster(Cluster topCluster)` |
| `void` | `setTotalClusters(double totalClusters)` |

**Methods inherited from class java.lang.Object**

`equals, getClass, hashCode, notify, notifyAll, toString, wait, wait, wait`

**Constructor Detail**

### Cluster

```
public Cluster(java.io.File floatArrayFile,
               java.lang.String name)
```

:   Initializing Constructor

---


### Cluster

```
public Cluster(Cluster one,
               Cluster two,
               double correlationCoefficient,
               java.lang.StringBuffer results)
```

:   Secondary Constructor


**Method Detail**

### getValues

```
public float[] getValues()
```

---


### getBottomCluster

```
public Cluster getBottomCluster()
```

---


### setBottomCluster

```
public void setBottomCluster(Cluster bottomCluster)
```

---


### getCorrelationCoefficient

```
public double getCorrelationCoefficient()
```

---


### setCorrelationCoefficient

```
public void setCorrelationCoefficient(double correlationCoefficient)
```

---


### getFloatArrayFile

```
public java.io.File getFloatArrayFile()
```

---


### setFloatArrayFile

```
public void setFloatArrayFile(java.io.File floatArrayFile)
```

---


### getName

```
public java.lang.String getName()
```

---


### setName

```
public void setName(java.lang.String name)
```

---


### getParentCluster

```
public Cluster getParentCluster()
```

---


### setParentCluster

```
public void setParentCluster(Cluster parentCluster)
```

---


### getTopCluster

```
public Cluster getTopCluster()
```

---


### setTopCluster

```
public void setTopCluster(Cluster topCluster)
```

---


### getTotalClusters

```
public double getTotalClusters()
```

---


### getNameLabel

```
public javax.swing.JLabel getNameLabel()
```

---


### setNameLabel

```
public void setNameLabel(javax.swing.JLabel nameLabel)
```

---


### setTotalClusters

```
public void setTotalClusters(double totalClusters)
```

---


### getLine

```
public java.awt.geom.Line2D getLine()
```

---


### setLine

```
public void setLine(java.awt.geom.Line2D line)
```

---


### getNameLabelCenterYCoordinate

```
public double getNameLabelCenterYCoordinate()
```

---


### setNameLabelCenterYCoordinate

```
public void setNameLabelCenterYCoordinate(double centerYCoordinate)
```

---


### getParentPoint

```
public java.awt.geom.Point2D getParentPoint()
```

---


### setParentPoint

```
public void setParentPoint(java.awt.geom.Point2D parentPoint)
```


---


|  |  |  |
| --- | --- | --- |
| **Overview** \| **Package** \| **Class** \| **Use** \| **Tree** \| **Deprecated** \| **Index** \| **Help** | |  |
| PREV CLASS   **NEXT CLASS** | **FRAMES**    **NO FRAMES**     **All Classes** |  |
| SUMMARY: NESTED \| FIELD \| CONSTR \| METHOD | DETAIL: FIELD \| CONSTR \| METHOD |  |


---
